# Are viruses associated with disc herniation? A clinical case series

**DOI:** 10.1186/s12891-020-3052-8

**Published:** 2020-01-14

**Authors:** B. F. Walker, A. J. Armson, M. A. O’Dea, J. R. White, C. R. P. Lind, P. R. Woodland

**Affiliations:** 1 Discipline of Psychology, Counselling, Exercise Science and Chiropractic, Murdoch University, Murdoch, Western 90 South Street, Murdoch, 6150 Australia; 2 Veterinary Virology, College of Science, Health, Engineering and Education, Murdoch University, Murdoch, Western Australia; 3 Australian Animal Health Laboratory, CSIRO, Geelong, Victoria Australia; 4 Neurospinal Department, St. John of God Hospital, Subiaco, Western Australia; 5 Neurosurgical Service of Western Australia, Sir Charles Gairdner Hospital, Nedlands, Western Australia; 6 Medical School, University of Western Australia, Nedlands, Western Australia; 7 Spinal Service, Department of Orthopaedics, Royal Perth Hospital, Perth, Western Australia

**Keywords:** Intervertebral disc herniation, Herpes virus, Low back pain

## Abstract

**Background:**

There is some limited evidence for the presence of viruses in herniated disc material including a previous case series that claimed to provide “unequivocal evidence of the presence of herpes virus DNA in intervertebral disc specimens of patients with lumbar disc herniation suggesting the potential role of herpes viruses as a contributing factor to the pathogenesis of degenerative disc disease”. This study has not been replicated. The objective of our study was to determine if viruses were present in herniated disc fragments in participants with a prior history of back pain.

**Methods:**

We recruited fifteen participants with a history of prior low-back pain prior to undergoing disc herniation surgery in the lumbar spine. Harvested disc samples were subject to next generation sequencing for detection of both RNA and DNA viral pathogens. Additionally, samples were analysed by a broadly reactive PCR targeting herpesviral DNA. Ethics approval was granted by the Human Research Ethics Committees of both Murdoch University, and St John of God Hospital, Western Australia.

**Results:**

Of the fifteen research participants, 8 were female. Mean age was 49.4 years (SD 14.5 yrs) with a range of 24–70 years. All participants had prior back pain with mean time since first ever attack being 8.8 years (SD 8.8 yrs). No samples contained significant DNA sequences relating to known human viral agents. Inconsequential retroviral sequences were commonly found and were a mixture of putative animal and human retroviral protein coding segments. All samples were negative for herpesvirus DNA when analysed by pan-herpesvirus PCR.

**Conclusions:**

This study found no viral pathogens in any intervertebral disc fragments of patients who had previous back pain and underwent discectomy for disc herniation and thus it is unlikely that viruses are associated with disc herniation, however given the contradiction between key studies enhanced replication of this experiment is recommended.

## Background

Back pain is a disabling musculoskeletal condition affecting almost everyone at some time [1]. Most of the cause of low back pain is unknown and accordingly is termed non-specific low back pain. However, some causes are known including disc herniation which can present with concurrent leg pain or sciatica from nerve root compression. In the majority of cases the offending disc is likely to have been degenerate. This degeneration may have been associated with trauma, genetics or aging. Some studies have shown associations between degenerative manifestations on lumbar magnetic resonance imaging (MRI) and low back pain [2] but the correlation is not certain [3]. Disc degeneration and herniation has been identified in 54 and 27% of people respectively with low back pain [2] but many with these changes do not experience continuing pain and it should be noted that not all disc herniation occurs in the context of degenerative disease. Indeed, degenerative changes do not always precede disc herniation [4].

Bacterial disc infection or “discitis” is a well-defined but uncommon presentation [5]. More recently bone oedema, known as modic changes have been studied and found to occur in 6% of the population but are also found in 35–40% of people with low back pain [6] . This same study found aerobic and anaerobic bacteria in the nuclear tissue of lumbar discs in patients undergoing spinal surgery for disc herniation which may be associated with the bone oedema [7]. Two recent studies found the presence of bacteria [8] and viruses [9] in the intervertebral discs of patients treated for lower back pain and both were postulated to be associated with disc herniation and/or degeneration. In the Alpantaki et al. study [9] the authors assessed the incidence of herpesviruses in intervertebral disc specimens from patients who underwent surgery for lumbar disc herniation. A polymerase chain reaction, nucleic acid hybridization assay was applied to screen for the DNA of eight different herpes viruses in 16 patients and two controls. DNA of at least one herpes virus was detected in 13 specimens (81.25%). Herpes simplex virus type-1 (HSV-1) was the most frequently detected virus (56.25%), followed by cytomegalovirus (CMV) (37.5%). In two patients, coinfection by both HSV-1 and CMV was detected. All samples, including two control specimens, were negative for HSV-2, varicella zoster virus, Epstein Barr Virus, and HSV 6, 7 and 8. There was an absence of an acute infection confirmed both at the serological and mRNA level. The authors concluded that these findings for the apparent presence of herpes virus DNA in intervertebral disc specimens of patients with lumbar disc herniation suggested a potential role for herpesviruses as a contributing factor in the pathogenesis of degenerative disc disease and subsequent herniation. However, it should be noted that the viruses present in the disc material may not have caused any pathology.

In summary, there is some limited evidence for the potential presence of viruses in herniated disc material but current suitably documented accounts are scant and further studies are needed. The aim of this project was to examine human vertebral disc fragments removed at routine discectomy surgery to look for the presence of potential viral pathogens using non-discriminatory whole genome sequencing. This technology is rapidly becoming the approach of choice for clinical investigations of diseases with unknown etiologies [10–12].

Null hypothesis: That herniated disc material from participants with previous low back pain will not contain viruses.

## Materials and methods

We recruited fifteen participants prior to undergoing disc herniation surgery in the lumbar spine. Inclusion criteria were: a history of low back pain preceding disc herniation, aged over 18 years, undergoing surgery for removal of disc material. Exclusion criteria were: immuno-compromised or currently ill with an infection.

Demographic questions were asked regarding age and sex. Direct questions were asked about length of time since first episode of troublesome back pain, location of worst pain (back or leg), and worst pain in the past month on a numerical rating scale (0–10). In addition we administered the Functional Rating Index (FRI), which contains 10 short item questions that measure pain and function of the spinal musculoskeletal system: eight refer to activities of daily living that might be adversely affected by a spinal condition; two refer to different attributes of pain. The Functional Rating Index (FRI) has been found to have good psychometric qualities acceptable for clinical and research use [13]. The FRI is scored out of 40 and averaged to a percentage. It estimates disability as follows, 0–20% = minimal disability, 21–40% = moderate disability, 41–60% = severe disability and 61% + = very severe disability.

With informed consent we used sections of vertebral disc fragments removed during surgical interventions from fifteen patients being treated for disc herniation. The sterile samples were immediately stored at 4 °C for no more than 48 h, before transport to Murdoch University and stored at − 80 °C. Following this, samples were prepared for next generation sequencing for detection of pathogens.

Intervertebral disc material nucleic acid was extracted using a Purelink Genomic DNA extraction kit (Invitrogen) according to the manufacturer’s instructions, with the modification to omit the RNAse treatment step. In order to capture potential RNA and DNA viral species for downstream NGS, total nucleic acid was converted to dsDNA via a combination of random priming and Klenow fragment based extension and PCR [14] . Briefly, samples were subject to reverse transcription using 1 μL primer NGS1random (CCTTGAAGGCGGACTGTGAGN_8_) at a concentration of 100 μM, 7 μL of purified sample, 10 μL of Protoscript II buffer (New England BioLabs) and 2 μL of Protoscript II First Strand cDNA enzyme (New England BioLabs) under the following reaction conditions: 25 °C for 5 min, 42 °C for 60 min and 95 °C for 3 min. Complementary strand synthesis was performed by adding 2 μL of primer NGS1random (10 μM concentration) and 1 μL of Klenow polymerase (Promega) and incubating the reaction at 37 °C for 1 h. Double-stranded DNA products were then amplified using primer NGS1 (CCTTGAAGGCGGACTGTGAG) at a final concentration of 1 μM and AmpliTaq Gold 360 mastermix (Life Technologies) under the following conditions: denaturation at 95 °C for 5 min, followed by 40 cycles of 95 °C for 1 min, 55 °C for 1 min, 72 °C for 1 min (increasing by 5 s per cycle), and a final extension step of 72 °C for 10 min. PCR product clean-up was performed using a Wizard SV gel and PCR kit (Promega). Following PCR clean up, 1 ng of DNA from each sample underwent library preparation and individual barcoding using a Nextera XT DNA library preparation kit (Illumina) according to the manufacturer’s instructions. Library sample quality control and concentration were analysed using a LabChip GX (Perkin-Elmer) and Qubit fluorometer (Invitrogen) respectively. Final libraries were pooled in equimolar amounts, and sequencing performed on an Illumina NextSeq using a mid-output 2 × 250 flowcell.

Total reads from each sample were mapped to the reference human genome (HG18; NCBI36 BioProject PRJNA31257) using Bowtie2 to remove host reads [15], and unmapped reads were collected for further analysis. Unmapped reads underwent de novo assembly using SPAdes [16] default assembly settings. Contigs were searched against the entire NCBI non-redundant protein database via blastx for homology to viral agents using DIAMOND [17] under default search settings with the exception to only return hits above an e-value cutoff threshold of 10^− 5^.

Based on the results reported by Alpantaki et al., a pan-herpesvirus nested PCR designed to amplify a 250 bp partial region of the DNA polymerase gene from a wide range of herpesviruses including human herpesviruses I – 6,was also performed using the primers developed by VanDevanter et al. [18]. Briefly Primary PCR mixtures contained two upstream primers (DFA, 5-GAYTTYGCNAGYYTNTAYCC-3; and ILK, 5-TCCTGGACAAGCAGCARNYSGCNMTNAA-3) and one downstream primer (KG1, 5-GTCTTGCTCACCAGNTCNACNCCYTT-3) in a multiplex format. Secondary PCRs were performed with upstream primer TGV (5-TGTAACTCGGTGTAYGGNTTYACNGGNGT-3) and downstream primer IYG (5-CACAGAGTCCGTRTCNCCRTADAT-3) under the same conditions used for the primary reaction. All reactions were run using Equine herpesvirus 1 and Crocodile herpes virus as positive controls, and products were electrophoresed on a 2% agarose gel containing 0.01% gel red stain. Ethics permission was granted by the Human Research Ethics Committee of both Murdoch University (2016/156) and St John of God Hospital, Subiaco (Reference 1074). Prospective participants were identified by the spine surgeon who asked their secretary to provide the patient with an information letter and a consent form. The surgeon did not approach the patient directly and the patient was assured that non-participation in no way altered their relationship with the surgeon or hospital. The handling of human tissue was in accordance with best practice and complied with the National Statement on Ethics of the National Health and Medical Research Council of Australia [19]. If pathological examination revealed any significant pathogen the patient and their surgeon were to be alerted immediately. Participants received the results of the pathological examination of the tissue with an explanation from the surgeon about the importance or non-importance of the findings.

## Results

Of the fifteen research participants, 8 were female and 7 male. Mean age was 49.4 years +/− 14.5 years (SD) with a range of 24 to 70 years. The first ever troublesome attack of back pain was a mean of 8.8 years previously (SD 8.8 years), the location of worst pain was in the leg for 11 cases and in the back for 3 cases (1 missing answer). The mean worst pain in the past month on a numerical rating scale was 7.9/10 (SD 1.6). The mean Functional Rating Index was 66.8% (SD 17.6) which indicates very severe disability overall. Samples from the 15 participants were processed separately through the library preparation pipeline. Samples returned an average of 9.9 million reads per sample before mapping to HG18 (range 7.4–13.8 million), and 3.4 million reads per sample after human genomic reads were removed. Following DIAMOND analysis of contigs and filtering for viral hits a large range of endogenous retroviral sequences were returned, however no samples contained any other significant hits to known human viral agents. While retroviral sequences were a mixture of putative animal and human retroviral proteins, the most prominent were multiple sclerosis associated retrovirus polyprotein sequences which were frequently detected in all samples. DNA from all samples was also subject to pan-herpesvirus PCR, however all samples were negative for herpesvirus DNA using this targeted approach. We compared observed and expected frequencies of our samples results. The expected number of virus positive samples were derived from the Alpantaki results of 13 virus positive samples from 16 specimens (~ 80%). Given this result, we expected to find 12 virus positive samples from our total of 15. Instead, we observed zero virus positive samples. The observed and expected results were entered into SPSS (Version 24.0.,0) and a Goodness of Fit test performed using a Chi-Square test combined with Fisher’s Exact Test. This resulted in a Chi-Squared value of 65.00 with 1 degree of freedom and a two-tailed *P* value of *P* < 0.000. The significant P value indicates that if the Alpantaki results (that generated the expected values) are correct, the probability of observing such a large discrepancy (or larger) between our observed and expected values with such a small P value is evidence that the data were not sampled from the distribution expected. It is noted that the chi-square calculations are only reliable when all the expected values are 5 or higher. This assumption is violated by our data, accordingly we used Fisher’s Exact test. Even so the *P* value may not be precise, but is highly indicative.

## Discussion

Our objective was to interrogate herniated disc samples for the presence of potentially pathogenic viruses. A positive result could act as evidence of concept that a putative viral agent may be associated with disc herniation. While we generally replicated the methods of Alpantaki et al. (2011) to address their findings that herpesviruses are present in the majority of disc herniations, we also applied non-targeted next generation sequencing technology to broaden the range of potential viruses found. This study failed to replicate the findings of Alpantaki et al. (2011), with no viral pathogens found. Alpantaki et al. found 56.25% of disc samples had herpes simplex virus type 1 (HSV1), and 37.5% had cytomegalovirus (CMV). HSV1 becomes latent within the trigeminal ganglia [20], and CMV within myeloid cells of the bone marrow [21], which indicates that latent virus is unlikely to be the reason for Alpantaki’s findings. The difference in results between this study and the Alpantaki study cannot easily be explained. If the prevalence of HSV or CMV were radically different between Greece and Australia this may be an explanation, especially if the prevalence was high in Greece and low in Australia. However, the overall age-adjusted seroprevalence of HSV-1 and HSV-2 in Greece was found to be 72.0 and 10.2%, respectively [22] and in Australia 76 and 12% respectively [23]. Regarding cytomegalovirus, the prevalence in a study of Greek mothers was 76% [24] (no recent national data found) while a national sero-survey in Australia demonstrated a prevalence of 57% [25]. Moreover, in both our study and the Alpantaki et al. study, general sample acquisition and case inclusion criteria were similar, and case numbers were also similar. The Alpantaki study utilized a RhyMA Test-Herpes screening kit for determination of the presence of herpesvirus DNA. The sensitivity and specificity of this test was not documented in the manuscript, and no data could be found online. However, while there is the possibility of a less than 100% level of specificity, it is unlikely that all samples reported were false positives and their study did confirm the specificity of the amplified, hybridized bands using nucleotide sequence analysis. In terms of the negative findings of our study, detecting viral sequences in genomic DNA using whole genome sequencing can be difficult, with host reads and other exogenous sequences generating greater than 99% of the read data. However, even after removal of host reads, an average of 3.4 million reads were present per sample, making it unlikely the presence of herpes virus DNA would not be detected. This was also confirmed by the use of a targeted pan-herpesvirus PCR, which also returned negative results. This PCR has been demonstrated to detect human herpesviruses 1–6 DNA, and numerous other herpesviruses including species as diverse as crocodile herpes virus [26]. In addition, this a nested PCR, which adds a significant increase in sensitivity to a standard PCR protocol or a hybridization based assay.

The presence of multiple endogenous retroviral sequences is not unexpected and reflects the historic integration of these agents into the human genome. While there may be some potential for these agents to be associated with spinal disease, investigating this is well outside the scope of this study, and would require a much larger study, inclusive of factors such as viral transcriptomic studies and in-situ visualization of viral antigens.

While multiple sclerosis associated retrovirus has been associated with disease, it is also common in clinically normal patients, and despite its name there is minimal support for causation of multiple sclerosis [27]. It has previously been shown that viral polymerase sequences are integrated into the genome of healthy humans [28], thus validating the findings in this study. In addition, the detection of these sequences does not indicate the presence of transcripts or replicating virus.

## Limitations of the study

We conducted a single arm study of 15 cases of intervertebral disc herniation in the lumbar spine and given that there are over 13,000 such operations each year in Australia alone [29] it is possible that we have missed detecting viruses due to the sample size. Nevertheless, Alpantaki et al. [9] were able to identify herpesviruses in 13 of 16 cases so while possible it appears unlikely that we would have missed at least one case in 15 consecutive instances. Nonetheless, given serology wasn’t performed in the current study, we would recommend that a population of patients seropositive for herpesvirus be included in future confirmatory studies.

A recent study has demonstrated significant alterations in mRNA expression of genes associated with proteoglycan and collagen synthesis in human nucleus pulposus cells when infected in vitro with herpes simplex virus 1 [30]. This is an interesting development demonstrating permissiveness of these cell types to viral infection and provides some further evidence that viral infection may have a role to play in disc degeneration. As for serology, repeat studies assessing mRNA expression in diseased tissues in combination with non-targeted viral detection will greatly add to our understanding of causation.

Finally, if there were major differences in distributions of degeneration in the herniated disc samples between the Alpantaki et al. study and this current study, it may go some way to explain the discrepancy between results.

## Conclusion

In conclusion, this study found no viral pathogens in any intervertebral disc fragments of patients who had previous back pain and underwent discectomy for disc herniation and thus failed to replicate the findings of Alpantaki et al. (2011). Based on this study the null hypothesis holds and while possible, it seems unlikely that viruses are associated with disc herniation. However, given the contradiction between the results of the two key studies replication of this experiment with the inclusion of seropositive and seronegative cohorts, along with transcriptome analysis, is recommended.

## Data Availability

Data availability results reported in this article can be found at the office of Professor Bruce Walker AM, Murdoch University, Murdoch, Western Australia 6150. The datasets used and/or analysed during this current study are available from the corresponding author on reasonable request.

## Abbreviations

CMV: Cytomegalovirus
DNA: Deoxyribonucleic acid
FRI: Functional rating index
HSV-1: Herpes simplex virus type-1 HSV-2 Herpes simplex virus type-2
MRI: Magnetic resonance imaging
mRNA: Messenger ribonucleic acid
PCR: Polymerase chain reaction
